# Multiplexed CRISPR/Cas9 Targeting of Genes Implicated in Retinal Regeneration and Degeneration

**DOI:** 10.3389/fcell.2018.00088

**Published:** 2018-08-21

**Authors:** Arife Unal Eroglu, Timothy S. Mulligan, Liyun Zhang, David T. White, Sumitra Sengupta, Cathy Nie, Noela Y. Lu, Jiang Qian, Lisha Xu, Wuhong Pei, Shawn M. Burgess, Meera T. Saxena, Jeff S. Mumm

**Affiliations:** ^1^Department of Ophthalmology, Wilmer Eye Institute, Johns Hopkins University School of Medicine, Baltimore, MD, United States; ^2^Translational and Functional Genomics Branch, National Human Genome Research Institute, Bethesda, MD, United States

**Keywords:** CRISPR/Cas9, zebrafish, retina, regeneration, retinal degeneration, large scale, multiplex, *rhodopsin*

## Abstract

Thousands of genes have been implicated in retinal regeneration, but only a few have been shown to impact the regenerative capacity of Müller glia—an adult retinal stem cell with untapped therapeutic potential. Similarly, among nearly 300 genetic loci associated with human retinal disease, the majority remain untested in animal models. To address the large-scale nature of these problems, we are applying CRISPR/Cas9-based genome modification strategies in zebrafish to target over 300 genes implicated in retinal regeneration or degeneration. Our intent is to enable large-scale reverse genetic screens by applying a multiplexed gene disruption strategy that markedly increases the efficiency of the screening process. To facilitate large-scale phenotyping, we incorporate an automated reporter quantification-based assay to identify cellular degeneration and regeneration-deficient phenotypes in transgenic fish. Multiplexed gene targeting strategies can address mismatches in scale between “big data” bioinformatics and wet lab experimental capacities, a critical shortfall limiting comprehensive functional analyses of factors implicated in ever-expanding multiomics datasets. This report details the progress we have made to date with a multiplexed CRISPR/Cas9-based gene targeting strategy and discusses how the methodologies applied can further our understanding of the genes that predispose to retinal degenerative disease and which control the regenerative capacity of retinal Müller glia cells.

## Introduction

Damage to specific retinal neuronal subtypes underlies several retinal degenerative diseases such as retinitis pigmentosa (RP), age-related macular degeneration and glaucoma. RP affects approximately 1 in 5,000 people worldwide and arises from numerous inherited conditions which lead to the progressive loss of rod photoreceptors, associated night blindness, tunnel vision, and eventual total loss of vision (Dias et al., 2017). Current treatment options have limited efficacy in slowing the progression or reversing the course of RP and related retinal degenerative disorders. It is therefore critical that the genes and signaling pathways which control degenerative and regenerative processes in the retina be identified. Such knowledge will support the development of therapies designed to maintain, or even restore, visual function in patients with debilitating retinal degenerative disorders.

The use of both forward and reverse genetics in model organisms has greatly facilitated the discovery of genes linked to disease pathology. Similarly, advances in next-generation sequencing technologies have led to marked increases in the number of candidate genes associated with human disease. More than 3,000 mutations in nearly 90 candidate genes have been associated with RP. Most of these genes are expressed in rod cells or the retinal pigment epithelium. However, because RP-associated genes affect multiple signaling pathways, a clear understanding of the molecular mechanisms leading to RP remains elusive. Large-scale targeting methods enable more comprehensive interrogation of gene function in animal models, thereby allowing assessment of the increasing number of candidate genes associated with disease phenotypes. Such studies can provide systems-level insights necessary to deconvolve the complexity of multifactorial signaling networks in disease pathogenesis and progression. In addition to facilitating mechanistic understanding of disease etiology, establishing animal models that more fully account for combinatorial genetic complexity of conditions such as RP will help enable the development of improved therapies.

The zebrafish (*Danio rerio*) is an attractive model for large-scale examinations of genotype to phenotype correlations. Early forward genetic screens in zebrafish succeeded in identifying numerous molecular pathways critical for development (Mullins et al., 1994; Solnica-Krezel et al., 1994; Driever et al., 1996; Haffter et al., 1996). In these and later visual function screens, zebrafish mutants displaying rapid onset retinal degeneration phenotypes were identified (for review see, Brockerhoff and Fadool, 2011; Link and Collery, 2015). Importantly, several mutants map to genes linked to retinal degeneration in humans (Starr et al., 2004; Stearns et al., 2007; Nishiwaki et al., 2008; Thiadens et al., 2009). These findings suggested orthologous gene function and confirmed the utility of zebrafish for retinal disease modeling. However, until recently, targeted mutagenesis methods were not available to directly investigate disease-implicated genome modifications in zebrafish. Widely applicable genome editing methods have filled this gap over the past decade, including: (1) Zinc Finger Nucleases (ZFNs; Doyon et al., 2008; Meng et al., 2008), (2) Transcription Activator-Like Effector Nucleases (TALENs; Huang et al., 2011; Bedell et al., 2012), and (3) Clustered, Regularly Interspaced, Short Palindromic Repeats (CRISPR; Hwang et al., 2013; Jao et al., 2013). The efficiency and ease of applying the CRISPR/Cas9 system, in particular, has ushered in the possibility of pursuing comprehensive reverse genetic screening in numerous model organisms.

Using the CRISPR/Cas9 technique, targeted double-strand breaks can be introduced in a genome resulting in short insertion and deletion (indel) mutations due to imprecise DNA repair mechanisms (Jinek et al., 2012; Cong et al., 2013; Mali et al., 2013). This system also facilitates precise editing of an endogenous locus when paired with an exogenous repair template (Ran et al., 2013; Wang et al., 2013). Since the first application of CRISPR/Cas9 mediated mutagenesis in zebrafish (Hwang et al., 2013), hundreds of targeted mutants have been generated, including numerous disease models (Baxendale et al., 2017; Küry et al., 2017; Van De Weghe et al., 2017; Prykhozhij et al., 2018). Multiplexed CRISPR/Cas9 methods have been developed (Cong et al., 2013; Mali et al., 2013), and adapted to the zebrafish system (Jao et al., 2013), which facilitate large-scale screening of mutations in genes of interest (Shah et al., 2015; Varshney et al., 2016a). This strategy can begin to fill a critical gap between modern -omics/bioinformatics platforms—which can implicate thousands of genes in a given biological process—and experimental testing capacities in model systems, particularly species such as zebrafish, flies, and worms which support large-scale phenotypic screening.

In addition to their amenability to large-scale screening, zebrafish have a robust capacity for retinal repair. Retinal regenerative medicine seeks to restore visual function to patients with degenerative diseases by replacing lost retinal cells. Two related strategies are being pursued, transplantation of retinal stem cells or stem cell-derived progeny (MacLaren et al., 2006; Schwartz et al., 2015; Shirai et al., 2016) and stimulation of endogenous regenerative capacities (Goldman, 2014; Jorstad et al., 2017; Elsaeidi et al., 2018). The innate reparative capacity of the zebrafish facilitates investigation of the latter. With respect to the eye, zebrafish can regenerate whole retinas (Sherpa et al., 2008), somal layers (Vihtelic and Hyde, 2000; Fimbel et al., 2007; Tappeiner et al., 2013; Powell et al., 2016), and even discrete retinal neuron subtypes (Ariga et al., 2010; Montgomery et al., 2010; Walker et al., 2012; Fraser et al., 2013). This remarkable regenerative ability arises from the major glial cell type of the retina, Müller glia (MG), which function as injury-induced stem cells in fish (Gorsuch and Hyde, 2014; Lenkowski and Raymond, 2014; Wan and Goldman, 2016).

MG cells are found in all vertebrates, but their responses to retinal injury and capacity for repair vary (Karl and Reh, 2010; Wilken and Reh, 2016). Unlike zebrafish, mammalian MG display no inherent regenerative capacities. Instead, retinal degeneration causes mammalian MG to undergo reactive gliosis, an inflammatory process that can exacerbate visual deficits (Bringmann et al., 2006). Zebrafish MG also undergo a gliosis-like response to retinal injury (Thomas et al., 2016), but this process resolves to allow proliferation. Intriguingly, mammalian MG retain the potential to act as stem cells. Cultured human MG can produce neurons (Lawrence et al., 2007) capable of restoring visual function when transplanted into retinal degeneration models (Singhal et al., 2012). Moreover, mammalian MG can be reprogrammed by expression of Lin28 and/or Ascl1 to generate new retinal neurons *in vivo* (Sanges et al., 2016; Jorstad et al., 2017; Elsaeidi et al., 2018). Harnessing this dormant reparative capacity for therapeutic benefit in humans will require a comprehensive understanding of the mechanisms regulating MG responses to cell loss, as well as MG-derived progenitor cell proliferation and differentiation.

To address this knowledge gap, we are using CRISPR/Cas9 to mutate genes implicated in retinal regeneration or linked to retinal degenerative disease. Multiplexed targeting methods are being applied to disrupt hundreds of genes implicated in regulating retinal regeneration, enabling a follow-on large-scale screen for genes required for rod photoreceptor regeneration. In addition, single- and multi-gene targeting methods are being used to disrupt RP-linked genes in an effort to create novel models of retinal degenerative disease, or to assess potential roles in regeneration in cases where degenerative phenotypes are lacking. Our initial results suggest that targeted large-scale CRISPR/Cas9 genome disruption strategies will be an efficient means of investigating genes and signaling networks implicated in controlling retinal disease pathogenesis and regulating the regenerative capacity of MG, an inducible retinal stem cell with unrealized therapeutic potential. We report here on our progress to date, focusing on multiplexed CRISPR/Cas9-based gene targeting strategies, genotyping and phenotyping screening strategies, mutagenesis success rates, and characterization of a new *rhodopsin* (*rho*) mutant model of autosomal dominant RP.

## Materials and methods

### Zebrafish husbandry

All studies were carried out in accordance with recommendations of OLAW for zebrafish studies and an approved Johns Hopkins University Animal Care and Use Committee animal protocol FI17M19. All fish were maintained at 28.5°C with a consistent 14:10 h light:dark cycle. All experiments were performed using either *royorbison (roy)/mpv17*^*a9/a9*^ or *Tg(rho:YFP-NTR)gmc500);roy/mpv17*^*a9/a9*^ fish (Ren et al., 2002; Walker et al., 2012; D'Agati et al., 2017).

### Preparation of synthetic single guide RNAS and Cas9-encoding mRNA

Synthetic single guide RNAs (sgRNAs) were designed to target regions of zebrafish candidate gene exons using CRISPRscan (http://www.crisprscan.org/). The targeted sequences for the *rho* gene are listed in Supplemental Table 1. sgRNAs for each target were generated using the cloning-free, oligonucleotide assembly method as previously described (Varshney et al., 2015). sgRNA template was *in vitro* transcribed using the HiScribe T7 High Yield RNA Synthesis kit (New England BioLabs) according to the manufacturer's instructions. The sgRNAs were precipitated using isopropanol/sodium acetate. For *cas9* mRNA synthesis, the zebrafish codon optimized Cas9-encoding plasmid, pT3TS-nls-zCas9-nls (Addgene plasmid #46757), was used as template (Jao et al., 2013). The template DNA was linearized by *XbaI* and purified using a QIAprep purification column (Qiagen). 500 ng linearized template was used to synthesize Cas9-encoding mRNA using the mMESSAGE mMACHINE T3 kit (Ambion) and recovered by lithium chloride precipitation.

### Microinjection and DNA extraction from embryos

For single gene-targeting, mutations were generated by co-injecting 300 pg of Cas9-encoding mRNA with 50 pg of one to three sgRNAs. Three sgRNAs were targeted to the 3′ end of the single exon of *rho*. For multiplexing and CRISPRscan-based sgRNA prioritization tests, we mixed six sgRNAs at 25 pg each with 300 pg of Cas9-encoding mRNA. Injected embryos were incubated at 28.5°C and euthanized at 48 hpf for DNA extraction. DNA was extracted from eight uninjected and eight injected embryos using Extract-N-Amp Tissue PCR Kit (Sigma) or using 50 mM NaOH/Tris-HCl (Meeker et al., 2007). Data for characterized sgRNAs, i.e., gene targets and *in vivo* mutagenic activities at target sites have been uploaded to CRISPRz (https://research.nhgri.nih.gov/CRISPRz/), an online database for sharing mutagenic efficiency information that enables reuse of validated sgRNAs and improved computational design (Varshney et al., 2016b).

### Primer design and fluorescent PCR for fragment separation by capillary electrophoresis

Fluorescent PCR primer sequences for *rho* sgRNAs are listed in Supplemental Table 1. PCR reactions were amplified using a universal, fluorescently-labeled M13F primer, an M13F gene-specific forward primer and a pig-tailed gene-specific reverse primer with either AmpliTaq-Gold (Life Technologies) as described previously (Carrington et al., 2015) or Phusion High-Fidelity DNA Polymerase (Thermo Fisher Scientific) in a final reaction volume of 10 μl. PCR products were mixed with the GeneScan 400HD ROX dye size standard (Life Technologies) by adding 10 μl of a 1:50 mix of 400HD ROX and HiDi-Formamide (Life Technologies) to 2.5 μl of PCR product. Samples were denatured at 95°C for 5 min and run on the Genetic Analyzer 3130xl using Pop-7 polymer. Data were analyzed for allele sizes and corresponding peak heights using the local Southern algorithm available in the Genescan and Genotyper software of the GeneMapper software package (Life Technologies). Allele sizes were used to calculate the size of indel mutations.

### F1 propagation and genotyping

*Tg*(*rho:YFP-NTR)gmc500*;*roy*/*mpv17*^*a9/a9*^embryos injected with CRISPR/Cas9 sgRNAs found to be effective at mutating their target sites were raised to adulthood and outcrossed to *Tg*(*rho:YFP-NTR)gmc500*;*roy/mpv17*^*a9/a9*^ fish. Four pools of four larvae resulting from each outcross were sacrificed, processed into genomic DNA (gDNA) and PCR amplified for analysis of inheritance of mutations that could be identified, as previously described, by capillary electrophoresis or by the presence of heteroduplex DNA formation on 3% metaphor agarose gels. Offspring of the putative founders (that gave rise to progeny with mutations in the desired genes) were raised to adulthood and fin clipped to isolate their gDNA. Individual F1 mutant alleles were confirmed by Sanger sequencing the PCR products amplified from fin clipped gDNA. The primers used to amplify *rho* alleles for Sanger sequencing are listed in Supplemental Table 1: *rho*-M13F_FORseq, *rho_*REVseq and *rho*_seq-nest. Genotyping *rho* mutants by resolution on a 3% metaphor (LONZA) electrophoresis gel was performed with the following primers: *rho*-M13F_FOR2 and *rho*-PIG_REV (Supplemental Table 1). For germline transmission analysis a minimum of three founders per injected group and 8-16 larvae per founder were screened.

### Confocal imaging

Mutant *Tg*(*rho:YFP-NTR)gmc500*;*roy/mpv17*^*a9/a9*^; *rho*^*djh503*+/−^ fish were outcrossed to homozygous *Tg*(*rho:YFP-NTR)gmc500*;*roy/mpv17*^*a9/a9*^ fish. Three larvae that appeared to be mutant based on their YFP expression levels and three that appeared to be wildtype were collected, embedded in 0.8% low melt agarose on their side and imaged daily from 3 to 7 days post-fertilization (dpf) with an Olympus Fluoview FV100 confocal microscope. A 20x water immersion objective and a 2.0x (day 3, 4 and 5) or 1.8x zoom (day 6 and 7) were used to image the eyes. Each eye was imaged using a Z-stack of 35–40 4 μm sections, and the resulting images were compiled into a maximum intensity projection image of the stack. Once the imaging was complete, the larvae were digested for gDNA extraction as previously described, PCR amplified and resolved on a 3% metaphor electrophoresis gel to confirm the mutant and wildtype phenotypes matched the expected genotype (Supplemental Figure 1). Imaging of cryosectioned and antibody-stained slides was performed using a 40x oil objective to obtain a Z-series of 3 images, each spaced 3 μm apart. The Z-series was then compiled into one image by applying the maximum intensity method in Fiji. The number of terminal deoxynucleotidyl transferase dUTP nick-end labeling (TUNEL) positive cells per eye was summed across three sections, each separated by 30 μm in the central region of the eye. Double-masked counts from three separate observers, of eight eyes per group, were averaged to determine the number of TUNEL positive cells. Mann-Whitney U tests were performed to evaluate the significance between wildtype and mutant groups.

### ARQiv assays

To quantify valproic acid (VPA)-induced inhibition of rod cell regeneration kinetics, *Tg*(*rho:YFP-NTR)gmc500*;*roy/mpv17*^*a9/a9*^ larvae were treated with 10 mM metronidazole (Mtz) for 24 hrs from 5 to 6 dpf to induce rod cell ablation. After Mtz washout and visual confirmation of rod cell loss, 12–16 larvae per condition were arrayed individually into wells of a 96-well plate containing either vehicle (E3 media), 100 or 200 μM VPA in E3 and incubated under standard conditions. At 9 dpf, larvae were anesthetized and processed for EYFP quantification using an Infinite M100PRO microplate reader (Tecan) as per established ARQiv protocols (Walker et al., 2012; White et al., 2016). Briefly, the limit of detection (i.e., “signal” cutoff) was defined as the mean of non-transgenic autofluorescent background plus three standard deviations. The reporter signal of transgenic fish was calculated as the sum of all scanned regions per well/fish that were equal to or greater than the limit of detection. The assay was repeated a total of three times.

To quantify developmental rod cell loss in 3–7 dpf *rho* mutants, 20 *Tg*(*rho:YFP-NTR) gmc500*;*roy/mpv17*^*a9/a9*^;*rho*^*djh503*+/−^ mutants, 20 *Tg*(*rho:YFP-NTR)gmc500*;*roy/mpv17*^*a9/a9*^ siblings and 24 non-transgenic *roy/mpv17*^*a9/a9*^ larvae were anesthetized and placed in individual wells of a black U-bottom 96-well plate (Greiner Bio-One). YFP signals were quantified as above. After each reading, all fish were washed out of anesthetic and recovered in PTU/E3 to allow longitudinal monitoring of YFP levels daily from 3 to 7 dpf. The genotype of each larva was confirmed after quantification. All data underwent log base 2 transformation prior to plotting. Two-sample *t*-tests followed by Bonferroni correction for multiple comparisons were performed to examine signal differences between groups on each day of the assay.

### Immunostaining

Four *Tg*(*rho:YFP-NTR)gmc500*;*roy/mpv17*^*a9/a9*^;*rho*^*djh503*+/−^ mutant larvae and four *Tg*(*rho:YFP-NTR) gmc500*;*roy/mpv17*^*a9/a9*^*;rho*^*djh503*+/+^ siblings were collected from 3 to 7 dpf. The head of each larva was fixed in 4% PFA at 4°C overnight, and the tail portion was collected for DNA extraction and genotyping (see genotyping section). After five PBS washes and 30% sucrose infiltration, larvae were embedded in OCT. Cryosections of 10 μm thickness were prepared using a Leica cryostat (CM 3050S) for immunofluorescence staining. Sections were washed with PBS, and blocked with 1x PBST (PBS containing 0.5% Triton X-100 and 5% goat serum). After an hour incubation at room temperature, PBST was removed and the sections were incubated in diluted primary antibody overnight. Antibodies were diluted in 1x PBST as indicated below. The next day, 1x PBST was used three times to thoroughly wash off the primary antibody. The secondary antibody was applied for 2 h at room temperature. All slides were washed once with PBST and mounted with VECTASHIELD antifade mounting medium with DAPI (Vector). The primary antibodies and concentrations used in this study were zpr1 (i.e., anti-Arr3a; 1:200), zpr3 (1:200), 1d1 (i.e., anti-Rho; 1:50) and 4c12 (1:100). The secondary antibody used was goat anti-mouse Alexa 647 (1:1,000). The zpr1 and zpr3 monoclonal antibodies were obtained from the Zebrafish International Resource Center (ZIRC). The antigen recognized by the zpr1 antibody is now known to be Arrestin3a, the zpr3 antigen is unknown. The antigen recognized by the 1d1 antibody (aka, Ab1-Rho) is Rhodopsin, the antigen recognized by 4c12 is unknown. Goat anti-mouse Alexa 647 was obtained from Invitrogen (cat. #C10640, lot #1911332). Dr. James M. Fadool kindly provided 1d1 and 4c12 monoclonal antibodies.

### Cell death detection

Cryosections were prepared as for immunostaining. Ten micrometer sections were stained using the *In Situ* Cell Death Detection Kit, TMR red (Millipore Sigma). All procedures followed the manufacturer's protocol. Briefly, sections were washed with 1x PBS, followed by permeabilization with 1x PBST containing 0.1% Triton X-100 and 0.1% sodium citrate on ice for 2 min. Slides were washed with PBS and incubated with labeling solution containing terminal deoxynucleotidyl transferase at 37°C for 1 h. Slides were washed with PBS once more and mounted with VECTASHIELD antifade mounting medium with DAPI (Vector).

## Results

### Target gene selection

To identify genes with potential roles in retinal regeneration, transcriptomic analyses of various retinal injury paradigms have been performed in zebrafish (Cameron et al., 2005; Kassen et al., 2007; Qin et al., 2009; Morris et al., 2011; Sifuentes et al., 2016). We were particularly interested in applying this approach to explore differences between cell-type specific retinal regeneration paradigms. Using the nitroreductase system of targeted cell ablation (Curado et al., 2007; White and Mumm, 2013), we induced the death of either bipolar interneurons (Ariga et al., 2010) or rod photoreceptors (Walker et al., 2012) and compared gene expression changes across 11 time points encompassing the loss and replacement of these two retinal neuron subtypes (Walker et al., manuscript in preparation). This study implicated more than 1,700 candidate genes in retinal neuron regeneration. Though insightful, an increasingly familiar problem of scale emerges with these types of studies; the sheer volume of genes implicated precludes comprehensive testing for potential roles in the biological process of interest.

To begin to address this issue, we are applying multiplexed CRISPR-based gene disruption strategies (Cong et al., 2013; Varshney et al., 2015, 2016a) to enable large-scale targeted mutagenesis screening. To select targets for this screen, a candidate list was generated from genes specifically upregulated in the first 24 h following induction of rod photoreceptor and/or bipolar cell ablation. Genes selectively upregulated following rod or bipolar cell loss were included to test the concept of cell-specific regenerative factors. This subset was further filtered to emphasize genes reported to be expressed in the eye or neuronal cell types and/or involved in regenerative pathways (based on ZFIN and PUBMED searches). In addition, several genes characterized as necessary for retinal regeneration in prior studies (e.g., *ascl1a*; Fausett et al., 2008) were included as controls. A parallel transcriptomic analysis focused on identifying genes required for hair cell regeneration in fish (Pei et al., 2018) provides a means of further testing the concept of cell-specific regenerative factors; i.e., genes required for the regeneration of discrete cell types or tissues. Comparisons between the two retinal neuron regeneration paradigms and the hair cell study revealed several shared genes. To broaden our investigation to factors that may be involved in neuronal regeneration more generally, an overlapping set of genes was added to our list of targets.

Finally, to begin to assess conservation of gene function, establish new retinal disease models, and explore relationships between neuronal degeneration and regeneration, we also targeted RP-linked genes. RP-associated genes were selected from the literature (Ferrari et al., 2011) and RetNet, an online database of retinal disease genes (https://sph.uth.edu/retnet/home.htm). Here, we present the initial results of a pilot study targeting zebrafish orthologs of five human genes linked to RP: *PDE6A, PDE6B, PDE6G, ABCA4*, and *RHO* (Table 1; note, *ABCA4* is also linked to cone-rod dystrophy). Due to genome duplication, three of the selected genes *(PDE6G, ABCA4*, and *RHO*) have two paralogs. Thus, this group included eight zebrafish homologs of five RP-linked genes. In summary, starting with a total of 1,789 candidates, our selection process narrowed the list to 320 gene targets.

**Table 1:** The five human RP-linked disease genes and eight corresponding zebrafish orthologs targeted in this study, their corresponding phenotypes and expression patterns in zebrafish and any available mutant, transgenic and morpholino-based zebrafish models.

**Human gene (OMIM ID)**	**Human disease pheotype-(Inheritence)**	**Zebrafish orthologs (Ensembl IDENSDARG00000-)**	**Zebrafish expression**	**Mutagen/MO/Tg (ZMP allele)**	**Mutation type**	**Zebrafish phenotype**
PDE6A (180071)	RP43(AR)	*pde6a (000380)*	Retina/Rod^a^	ENU (sa14581)^f^	NS	NR
PDE6B (180072)	CSNB2(AD); RP40(AR)	*pde6b (011671)*	Retina/Rod^a^	MO1-pde6b^g^	N/A	No rod cell death
PDE6G (180073)	RP57(AR)	*pde6ga (056791)*	Retina/Rod^b,c^	ENU (sa31321)^f^	ESS	NR
		*pde6gb (101984)*	Retina/Rod^b,c^	NR	N/A	N/A
ABCA4 (601691)	CRD3(AR) with VE; RP19(AR); STDG1(AR); AMD2(AD)	*abca4a (057169)*	NR	*La022972Tg-Tg(nLacz-GTvirus)^*h*^* ENU (sa11328)^f^	TgI ESS	NR NR
		*abca4b (062661)*	NR	ENU (sa16259; sa16600; sa19702; sa19703; sa19704)f ENU (sa19705; sa31266)^f^	NS ESS	NR NR
RHO (180380)	CSNB1(AD); RP4(AR or AD); RPA (AR or AD)	*rho (002193)*	Retina/Rod^d^	*zf355Tg-Tg(rho:adey2b-rho)^*g*^*	TgI	Photoreceptor cell death
				*Tg(XOPS-mCFP)^*i*^*	TgI	Rod cell death
				ENU (sa21401; sa31692)^f^	NS	NR
		*rhol (070666)*	Retina/Rod^e^	ENU (sa21885)^f^	NS	NR

*Databases searched included OMIM, ZFIN, ZMP and ENSEMBL. AR, autosomal recessive; AD, autosomal dominant; AMD, age-related macular degeneration; CSNB, congenital stationary night blindness; CRD, cone-rod dystrophy; ENU, N-ethyl-N-nitrosourea; ESS, Essential splice site mutation; LCA, leber congenital amaurosis; MO, morpholino; N/A, Not Applicable; NR, Not Reported; NS, Nonsense mutation; RP, retinitis pigmentosa; RPA, retinitis punctata albescens; STDG, stargardt disease/Fundus flavimaculatus; Tg, transgene; TgI, Transgenic Insertion; VE, variable expressivity; ZMP, zebrafish mutation project*.

a *Nishiwaki et al. (2008)*.

b *Thisse and Thisse (2004)*.

c *Lagman et al. (2016)*.

d *Becker et al. (1998)*.

e *Morrow et al. (2011)*.

f *Busch-Nentwich et al. (2013)*.

g *Nakao et al. (2012)*.

h *Wang et al. (2007)*.

i *Morris et al. (2005)*.

### Multiplex strategy

To increase the chances of recovering loss of function mutations in candidate genes, we designed two synthetic single guide RNAs (sgRNAs) targeting early but separate exons in regeneration-implicated genes (except for smaller genes where two different positions in an early exon were targeted) and one to four sgRNAs targeting RP-linked genes using CRISPRscan (Moreno-Mateos et al., 2015). In all, we designed sgRNAs targeting 614 sites in 312 regeneration genes and 14 sites in 8 RP-linked genes. For the regeneration-implicated gene set, we multiplexed 6 sgRNAs to concurrently target 3 genes per injection, resulting in a total of ~100 pooled founder groups. For the RP-associated gene set, we multiplexed sgRNAs targeting a single gene per injection (except for *pde6ga* and *pde6gb* which were targeted together). Thus far, 342 sgRNAs have been generated targeting 191 genes and 57 pooled groups targeting 158 genes have been injected (Table 2). Data regarding the specific genes targeted and our success in mutagenizing target loci (i.e., somatic and germline activity of injected sgRNAs) has been uploaded to the CRISPRz database (https://research.nhgri.nih.gov/CRISPRz/) and will continue to be as verified results become available. CRISPRz provides a forum for sharing data on sgRNA activity, e.g., mutagenic efficiency, which enables reuse of validated sgRNAs and improved computational design (Varshney et al., 2016b).

**Table 2:** **Mutagenic efficiencies observed in injected F0 embryos and F1 founder screens**.

			**Injected screen results**				**Founder screen results**			
	**Genes targeted**	**Injections (gene # targeted)**	**Mutated genes assayed**	**Target amplicons (%)**	**Target genes assayed**	**Mutated genes (%)**	**Potential founders screened**	**Founders identified (%)**	**Mutated amplicons (%)**	**Mutated genes (%)**
RP-linked targets	8	7 (8)	14	12 (86%)	8	7 (88%)	34	22 (65%)	14 (100%)	8 (100%)
Regeneration targets	312	50 (150)	120	95 (79%)	91	84 (92%)	41	26 (63%)	27 (75%)	20 (83%)

Percentages reported are relative to the total number assayed or screened, to date.

### Targeting efficiency

To evaluate the mutagenic activity of sgRNAs, genomic DNA (gDNA) was extracted from injected larvae at 48 h post fertilization (hpf) and assayed either by resolution of heteroduplex DNA formation in PCR amplicons (Nagamine et al., 1989) using standard 3% agarose gel electrophoresis, or by resolution of fluorescent PCR products using capillary electrophoresis (Carrington et al., 2015; examples shown in Supplemental Figure 2). Mutagenic activity of sgRNAs were calculated as previously described (Carrington et al., 2015) and reported on the CRISPRz site. We obtained data from 134 targeted genomic sites. For the regeneration set, this analysis revealed that 79% of the assayed amplicons (95 of 120) and 92% of the targeted genes (84 of 91) were successfully mutated (Table 2). For RP-linked targets, 86% of targeted regions (12 of 14) and 88% of targeted genes (7 of 8) were successfully disrupted.

In cases where multiple sgRNAs were targeted to a single exon, the region containing both targets was often amplified by the same PCR primers. In these cases, gel or capillary electrophoresis does not allow the distinction between which sgRNAs mutate their target, thus we can only assay differences in amplicon sizes that could result from mutation at either site. As a result, the amplicon mutation efficiency rates reported are an estimate of *in vivo* sgRNA target efficiency. We observed a slightly higher mutation rate for assayed amplicons for RP-associated targets (86%)—where single gene targeting strategies were largely used (except for co-targeting of *pde6ga* and *pde6gb*)— than for multiplexed regeneration gene targets (79%). However, our ability to identify mutations in RP-associated targets (88%) was slightly lower than for multiplexed regeneration targets (92%). This data demonstrates that multiplexing did not have a negative effect on our ability to mutate targeted genes.

To test for germline transmission of CRISPR/Cas9-induced mutations, we raised injected embryos to adulthood and outcrossed them to *Tg(rho:YFP-NTR)gmc500;roy/mpv17*^*a9/a9*^ fish (hereafter, rho:YFP-NTR). gDNA was isolated from the progeny of each founder outcross and analyzed for indel mutations as described above. Of 41 initial potential founders screened in the regeneration gene set, 26 (63%) were found to pass mutations on to their progeny. Progeny of these founders inherited mutations in 1–3 of the targeted genes in each injection group and 1–6 mutant alleles per sgRNA target site (Supplemental Tables 2, 3 and Supplemental Figure 3). Of 34 successfully mated potential founders in the RP-linked set, 22 (65%) exhibited germline transmission. Despite incomplete mutagenic efficiency for several sgRNAs, our strategy of multiplexing and utilizing at least two sgRNAs per gene target resulted in the recovery of mutations in 83% of screened regeneration-implicated gene targets and 100% of the RP-linked gene targets (Table 2). To date, we have confirmed mutagenic activity in at least 131 of 177 sgRNAs (74%, assuming only one of two sgRNA target sites were mutated when two were simultaneously assayed; Supplemental Data File 1, see also CRISPRz site).

### Predictability of CRISPRscan

**Figure 1:**
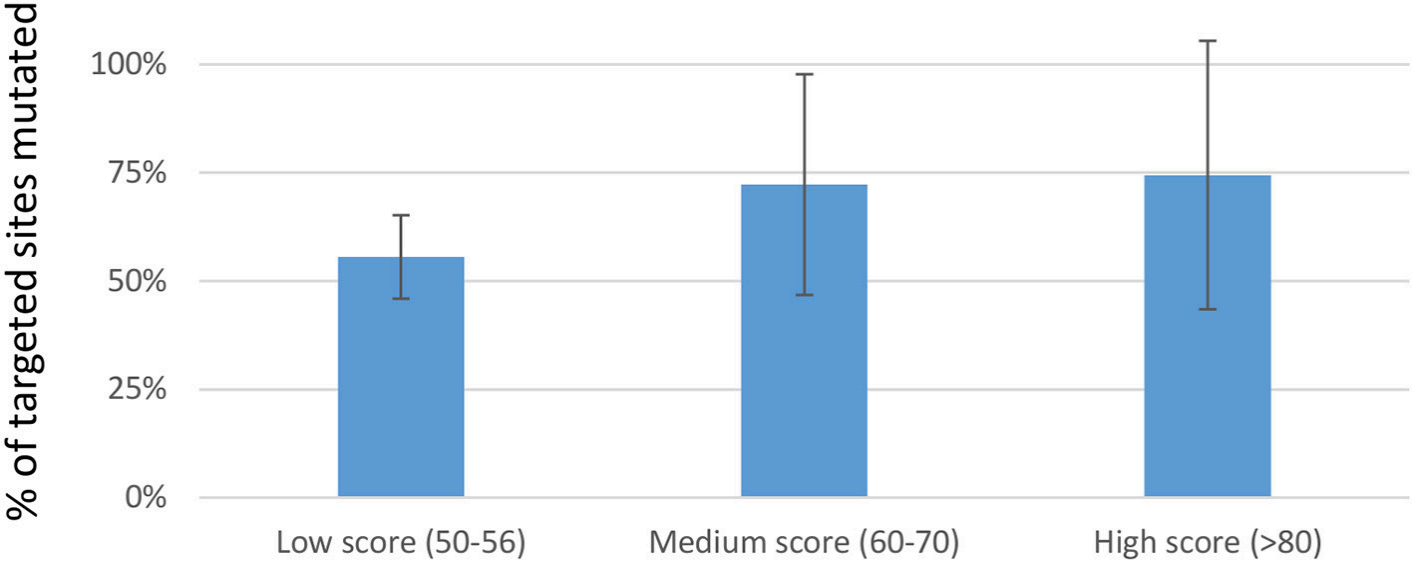
Percentage of targets mutagenized when multiplexed sgRNAs where separated into sets based on CRISPRscan score. For each set, three pools of 6 sgRNAs were prepared. Each pool of 6 sgRNAs was co-injected with Cas9-encoding mRNA into 1-cell embryos. Data is represented as the mean ± sd of targets mutated in each set. Although a trend toward improved mutagenic efficiency is evident, no statistically reproducible differences were found.

We next investigated whether *in silico* methods for rating sgRNA mutational efficiency could be used to eliminate the time and cost associated with evaluating hundreds of sgRNAs prior to their use in multiplexed applications. Various algorithms have been developed for predicting sgRNAs mutational efficacy and off-target effects. Prior studies suggest a reasonable correlation between CRISPRscan scores and mutagenic efficiency (Doench et al., 2014; Moreno-Mateos et al., 2015; Haeussler and Concordet, 2016; Albadri et al., 2017). We therefore asked whether CRISPRscan scores correlated with mutagenic efficacy in our hands. A sampling of sgRNAs was divided into three sets based on their CRISPRscan score; low (50–56), medium (60–70), and high (>80). Three pools of six sgRNAs were prepared for each set and co-injected with Cas9-encoding mRNA into one-cell zebrafish embryos. Mutagenic efficiency was evaluated by fluorescent PCR and capillary electrophoresis. Consistent with previous studies, we found a trend of increased indel frequency for sgRNAs with higher CRISPRscan scores. Data from 54 target sites showed an average of 77% targets mutagenized for high score sgRNAs, 72% for medium, and 56% for low (Figure 1). These results support the use of the CRISPRscan algorithm to prioritize sgRNAs for multiplexing injections in zebrafish based on score. In particular, sgRNAs scoring >60 were found to have a relatively high likelihood of successful targeting (>70% on average) and would be generally more useful for multiplexed targeting in zebrafish.

### Phenotyping strategy

To facilitate phenotyping, all pooled sgRNAs were injected into the *rho:YFP-NTR* background in which fluorescently-labeled rod photoreceptors can be selectively ablated (Walker et al., 2012). The *roy orbison (roy/mpv17*^*a9/a9*^*)* mutant line facilitates *in vivo* visualization and quantification of retinal cells expressing fluorescent proteins (Mumm et al., 2006). When combined with a fluorescence microplate reader assay we developed for rapid quantification and large-scale screening of reporters in living fish, termed ARQiv (Automated Reporter Quantification *in vivo*; Wang et al., 2015; White et al., 2016), the *rho:YFP-NTR* transgenic line allows the kinetics of rod cell loss and regeneration to be quantified longitudinally (Walker et al., 2012), thereby enabling detection of developmental and regeneration-modulating phenotypes. To mock-up regeneration-deficient phenotypic data, we impeded rod cell regeneration kinetics using a chemical inhibitor, e.g., pre-treatment with dexamethasone (White et al., 2017). In an ongoing AQRiv-based chemical screen for modulators of rod cell regeneration, we identified valproic acid (VPA) as an inhibitor (Supplemental Figure 4). To model an autosomal recessive phenotype, control and VPA-inhibited datasets were combined at a 3:1 ratio. Computational methods can then be used to predict sample size requirements (via power analysis) and to test real-time data processing algorithms for flagging clutches with potential regeneration-deficient phenotypes—similar to methods we established for zebrafish high-throughput assay development (White et al., 2016). This analysis suggests clutch sizes of ~100 will be adequate for detecting a 50% reduction in rod cell regeneration kinetics in 25% of offspring from genotyped F1 crosses, and that Hartigan's dip test statistic for unimodality (Hartigan and Hartigan, 1985; “diptest” package in R) will be useful for flagging clutches of interest.

### RP-linked mutant characterization

**Figure 2:**
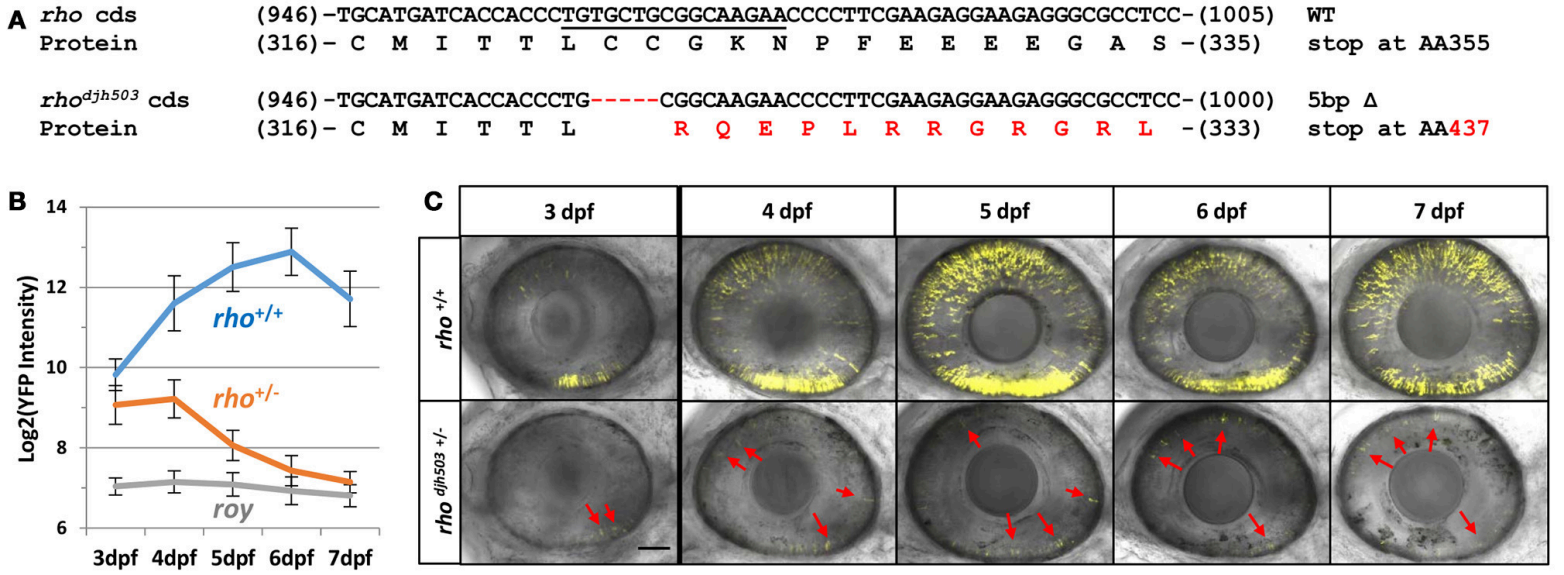
Genotype and phenotype of the CRISPR/Cas9-induced *rho*^*djh503*^ mutation. **(A)** Sequence of the wildtype *rho* coding sequence targeted by CRISPR/cas9 (sgRNA sequence underlined) and the location of the 5 bp deletion in the *rho*^*djh503*^ mutant allele. The amino acid sequences predicted to result from these open reading frames are shown below the nucleotide sequence with the altered sequence highlighted in red. **(B)** YFP signal intensity changes in *rho*^+/+^ fish and *rho*^*djh503*+/−^ mutant fish from 3 to 7 dpf (±sd). Daily fluorescence microplate readings of *rho*^+/+^ fish and *rho*^*djh503*+/−^ mutants in the *rho:YFP-NTR* background. The *roy* control group have no YFP-expressing transgene. Pairwise comparisons (i.e., *T*-test followed by Bonferroni correction for multiple comparisons) between per day data points produced *p*-values of ≤ 0.0005. **(C)** Composite maximum intensity projection images of confocal Z-stacks taken of the eyes of wildtype and mutant fish from 3 to 7 dpf. 3, 4, and 5 dpf images were taken with a 2x zoom while the 6–7 dpf images were taken with a 1.8x zoom. Several of the photoreceptors present in the mutant retinas are indicated by red arrows. The scale bar represents 50 μm in the 3–5 dpf images and 55.6 μm in the 6 and 7 dpf images. AA, amino acid; bp, base pair; cds, coding sequence; dpf, days post-fertilization; WT, wildtype; YFP, yellow fluorescent protein.

To begin to characterize RP-linked gene mutants, we assayed progeny from genotyped F1 mutants for RP-like phenotypes; i.e., early loss of rod photoreceptors. As proof of principle, we isolated a novel mutation in *rhodopsin (rho)*, which encodes the G-protein coupled receptor necessary for phototransduction in rod cells. Mutations in *rho* are responsible for 30% of the autosomal dominant cases of RP patients (Al-Maghtheh et al., 1993). The *rho*^*djh503*^ allele identified here contains a 5 base pair (bp) deletion in the targeted 3′ coding sequence of the gene [c.(964_968delTGCTG)]. This frameshift is predicted to alter the post-translationally palmitoylated cysteine at position 322 to an arginine [p.(Cys322Argfs^*^116)] and create an elongated protein that terminates at amino acid 437 rather than position 357 (Figure 2A and Supplemental Figure 1). This would result in a Rho protein with significant alteration of its cytoplasmic tail, an area necessary for trafficking from the inner to outer segment of rod photoreceptors (Sung et al., 1994). Rho proteins lacking this cytoplasmic tail lead to more aggressive rod degeneration in humans and model systems (Sandberg et al., 1995; Berson et al., 2002; Feng et al., 2017).

To characterize the effects of the *rho*^*djh503*^ mutation on the development and survival of rod photoreceptor cells, *rho*^*djh503*+/−^ fish were outcrossed to *rho:YFP-NTR* fish. As early as 3 days post-fertilization (dpf), with the emergence of YFP expression in rod cells, there was a noticeable difference in the level of YFP observed between *rho*^*djh*503+/−^ mutants and *rho*^+/+^ siblings using stereo fluorescence microscopy. Changes in YFP expression were quantified from 3 to 7 dpf in *rho*^*djh503*+/−^ mutants and *rho*^+/+^ siblings using our fluorescence microplate reader assay (Walker et al., 2012). Mutant *rho*^*djh503*+/−^ larvae exhibited a gradual loss of rod cells, while rod cell numbers increased over time in *rho*^+/+^ siblings (Figure 2B). By 7 dpf, detection of YFP signals in *rho*^*djh503*+/−^ mutants approached non-transgenic background levels. Time series confocal microscopy of *rho*^*djh503*+/−^ mutants and *rho*^+/+^ siblings from 4 to 7 dpf confirmed the progressive loss of YFP-expressing rod cells in mutant larvae (Figure 2C). These results indicated that the *rho*^*djh503*^ mutant phenotype is inherited in an autosomal dominant manner similar to many human *RHO* mutations (Sung et al., 1993).

**Figure 3:**
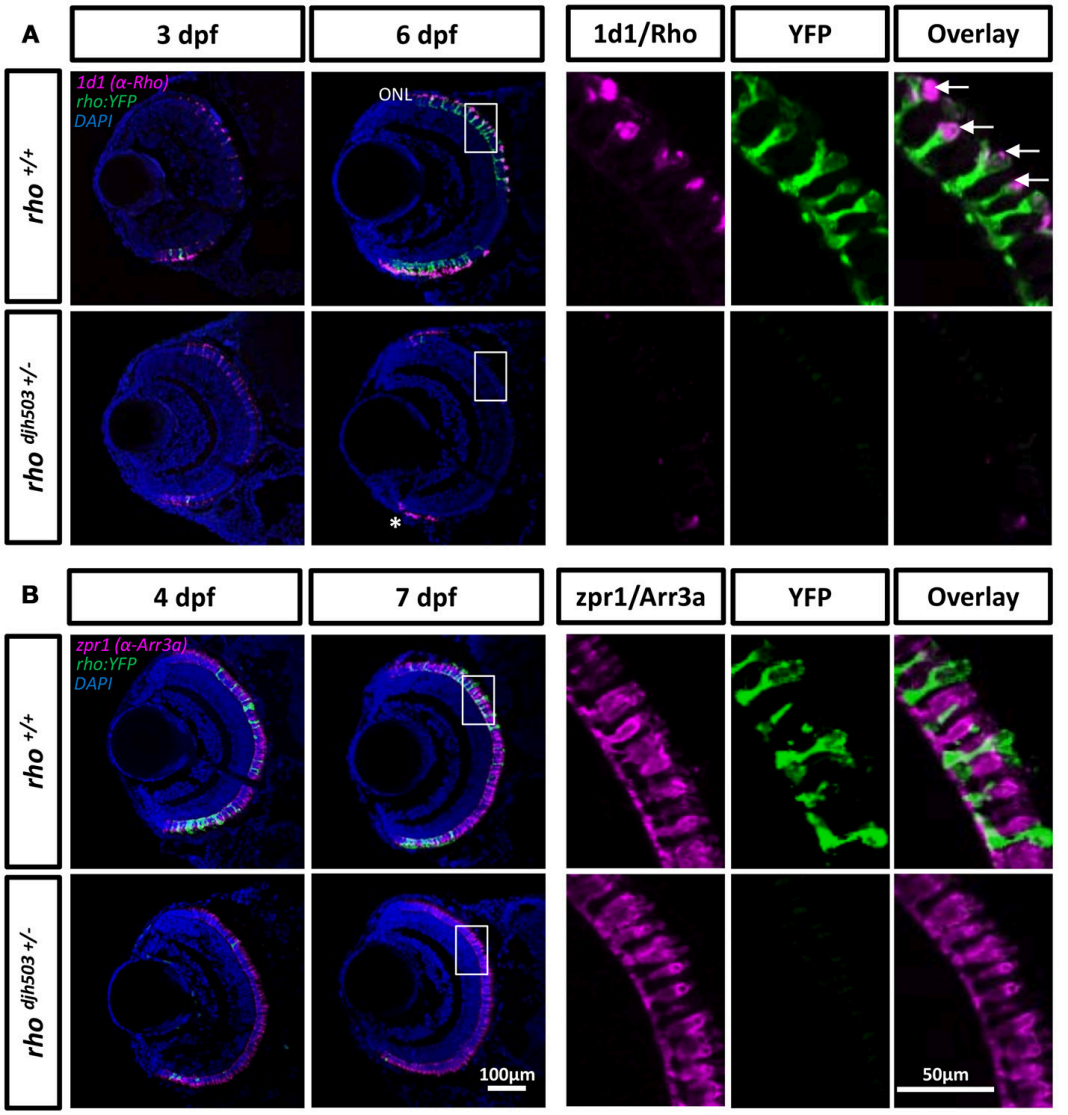
Rod but not cone photoreceptors are reduced in *rho*^*djh503*+/−^ mutants. **(A)** The 1d1 antibody (magenta), which recognizes *Rhodopsin*, labels the outer segments of wildtype rod cells. The labeling pattern is similar in both wildtype and mutants at 3 dpf. At 6 dpf, 1d1 antibody labeling is restricted to the proliferating marginal zone in *rho*^*djh503*+/−^ mutants (*), but remains throughout the ONL in wildtype retinas. The white boxed region in the 6 dpf images are enlarged in the panels to the right showing that the 1d1 antibody labels YFP-expressing rod cells (green, arrows). **(B)** The zpr1 (magenta) antibody labeled cone cells do not show an obvious difference between wildtype and mutant retinas at 4 and 7 dpf. The boxed region in the 7 dpf images is enlarged to the right showing no overlay between zpr1 antibody staining and YFP-expressing rod cells (green). DAPI (blue) was used to stain cell nuclei. Arr3a, Arrestin 3a; DAPI, 4′,6-diamidino-2-phenylindole; dpf, days post-fertilization; ONL, outer nuclear layer; *rho, rhodopsin*; Rho, Rhodopsin; YFP, yellow fluorescent protein.

To confirm the loss of rod photoreceptors in *rho*^*djh503*+/−^ mutants, immunostaining was performed using two rod-specific antibodies: 1d1, which labels Rhodopsin (Hyatt et al., 1996), and 4c12 which labels an unknown epitope on rod cells (Morris et al., 2005). At 3 dpf, both antibodies detected sparse numbers of emerging YFP-expressing rod photoreceptors in the outer nuclear layer (ONL, where photoreceptors reside) of *rho*^*djh503*+/−^ mutants and *rho*^+/+^ siblings (Figure 3A; Supplemental Figure 5A). The number of antibody labeled rod cells increased in wildtype larvae by 6–7 dpf. Conversely, mutant retinas exhibited almost no antibody staining in the ONL at 6–7 dpf. However, labeling was evident in the ciliary marginal zone where neurogenesis is ongoing in zebrafish (Figure 3A; Supplemental Figure 5A). The time-dependent reduction of rod-specific antibody staining in *rho*^*djh503*+/−^ mutants suggests that rod cells form normally during early developmental stages (1–3 dpf), but are rapidly lost over the next 4 days.

In RP disease progression, rod cell death typically precedes cone photoreceptor loss. To determine if cone cells were also affected in *rho*^*djh503*+/−^ mutants, immunostaining with zpr1 and zpr3 monoclonal antibodies was performed on 4–7 dpf larvae. Staining with zpr1, which labels cone photoreceptors (Larison and Bremiller, 1990; note, the antigen is now known to be Arrestin3a), did not show an obvious difference between *rho*^*djh503*+/−^ mutants and *rho*^+/+^ siblings, indicating that cone cells remain unaffected during early development (Figure 3B). Staining with zpr3, which labels both cone and rod photoreceptors (Larison and Bremiller, 1990; antigen unknown), was decreased in *rho*^*djh503*+/−^ mutants compared to *rho*^+/+^ siblings (Supplemental Figure 5B), demonstrating that rods but not cones are rapidly lost in *rho*^*djh503*+/−^ mutants.

**Figure 4:**
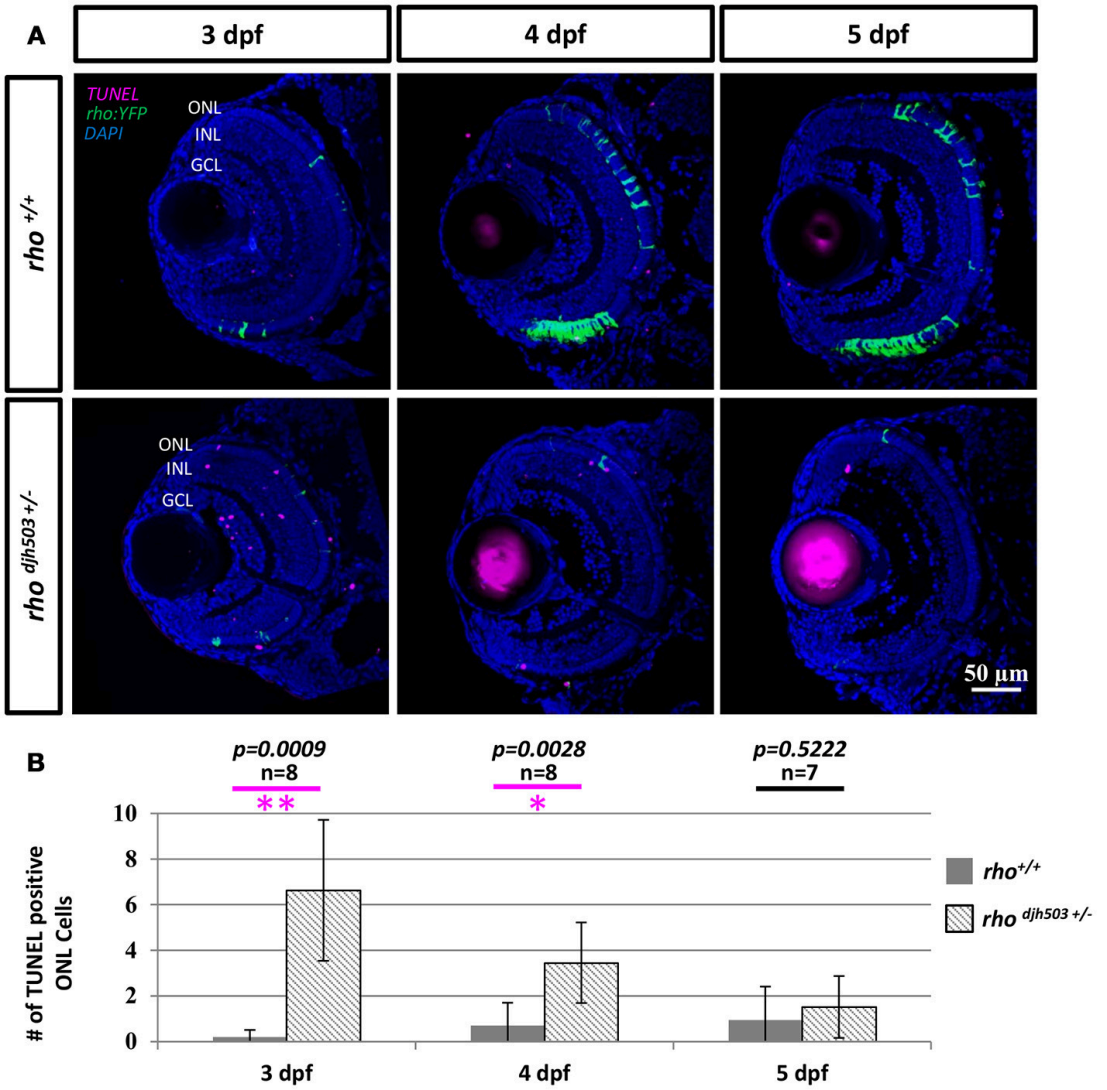
The number of apoptotic cells in the ONL is increased in *rho* ^*djh*503*c*+/−^ mutant retinas at early developmental stages. **(A)** Representative images of TUNEL stained retinas from 3 to 5 dpf. In wildtype retinas, TUNEL positive cells (magenta) were rarely seen in the ONL from 3 to 5 dpf. In contrast, TUNEL positive cells were frequently detected in *rho*^*djh503*+/−^ mutants at 3 and 4 dpf, but only occasionally at 5 dpf. Rod cells are labeled by YFP (green). **(B)** Quantification of TUNEL positive cells in the ONL. The number of TUNEL positive cells was significantly higher in *rho*^*djh503*+/−^mutants than in wildtype at 3 and 4 dpf, but not 5 dpf. Mann-Whitney test *p*-values: **p < 0.001, *p < 0.01. Sample size (n) of each condition is provided. DAPI (blue) was used to stain nuclei. dpf, days post-fertilization; GCL, ganglion cell layer; INL, inner nuclear layer; ONL, outer nuclear layer; *rho, rhodopsin*; TUNEL, terminal deoxynucleotidyl transferase dUTP nick-end labeling.

To investigate whether the decreased number of rod cells in *rho*^*djh503*+/−^ mutants was from apoptosis, TUNEL staining was conducted in 3–5 dpf larvae (Figure 4A). TUNEL positive cells were only occasionally observed in the ONL of *rho*^+/+^ larvae (3 dpf: 0.2 ± 0.3; 4 dpf: 0.7 ± 1.0; 5 dpf: 1.0 ± 1.5). In contrast, an increase in TUNEL positive cells was observed in the ONL of *rho*^*djh503*+/−^ mutants at 3 dpf (6.6 ± 3.1) and 4 dpf (3.5 ± 1.8; Figure 4B). By 5 dpf, there were no appreciable differences in TUNEL staining between *rho*^*djh503*+/−^ and *rho*^+/+^ larvae. The increased number of TUNEL positive cells in the ONL of *rho*^*djh503*+/−^ mutants supports the idea that rods develop initially, but undergo cell death shortly after differentiating such that almost no rods are evident by 7 dpf. An increased number of TUNEL positive cells was also seen in the inner nuclear layer (INL) and ganglion cell layer (GCL) in *rho*^*djh503*+/−^ mutants at 3 dpf. The implications of this observation are unclear. Overall, these results indicate that our mutagenesis strategy and *rho:YFP*-based transgenic screening assays are sufficient to identify mutants that exhibit loss of rod photoreceptors and provide potential new models of degenerative retinal disease.

## Discussion

Next-generation sequencing and multiomics technologies have greatly enhanced understanding of disease genetics and expanded investigatory perspectives from singular factors and linear pathways to a systems level of interacting molecular networks. While these methodologies provide data-rich resources for advances in biostatistics and mathematical modeling, a sizeable gap exists between the volume of information produced and the ability to experimentally test the predictions generated. CRISPR/Cas9-based targeted genome modification technologies provide a means of bridging this divide by enabling the creation of new disease models and targeted genetic screens on a large scale. Importantly, this breakthrough technology brings the power of reverse genetics to an ever-expanding number of model species. In turn, this broadens investigations of evolutionary conservation at the molecular level and allows inherent strengths of different model species to be brought to bear on important biological paradigms. Here, we present data that further supports the use of multiplexed CRISPR/Cas9-based mutagenesis strategies in the zebrafish as a reliable and rapid means of targeting genes of interest on scales that begin to match “big data” science (Jao et al., 2013; Li et al., 2013; Mali et al., 2013). We provide evidence of the utility of this approach for creating novel disease models based on GWAS-derived gene sets. Follow-on studies will focus on leveraging these resources for a guided, reverse genetic screen for genes required for retinal regeneration, as recently exemplifed in a CRISPR/Cas9-enabled screen for genes required for hair cell regeneration (Pei et al., 2018).

### CRISPR/Cas9 sgRNA targeting efficiency

Several algorithms have been developed to identify and predict which sgRNA targets will be efficiently mutated (Sander et al., 2010; Moreno-Mateos et al., 2015; Labun et al., 2016). The mutagenic efficiency of an sgRNA is determined by a variety of factors including chromatin structure, nucleosome positioning, DNA accessibility and sgRNA sequence-specific features (Horlbeck et al., 2016; Isaac et al., 2016; Thyme et al., 2016; Jensen et al., 2017). Nucleotides at both PAM-distal and PAM-proximal regions of the sgRNA, GC percentage and melting temperature of the target site, secondary structure of the sgRNA and the tracrRNA sequence and binding sites for epigenetic factors have been shown to influence sgRNA activity (Doench et al., 2014; Chari et al., 2015; Thyme et al., 2016; Yuen et al., 2017). In this study, we observed a trend of increased mutagenesis rates for sgRNAs with higher CRISPRscan scores. This suggests that assaying mutagenic efficiency of high scoring sgRNAs in injected embryos is not absolutely necessary. However, drawing conclusive correlations between the predictive scores of current *in silico* sgRNA target design algorithms and mutagenic efficiency of sgRNAs *in vivo* remains difficult (Moreno-Mateos et al., 2015; Haeussler and Concordet, 2016; Albadri et al., 2017). Further improvement of efficiency prediction algorithms will benefit large-scale mutagenesis efforts.

### Multiplexing CRISPR/Cas9 mutagenesis for large-scale genetic screening

The zebrafish system has facilitated unbiased genetic dissections of retinal development and visual function (Fadool and Dowling, 2008; Link and Collery, 2015; Stenkamp, 2015). However, of thousands of genes implicated in retinal regeneration (Morris et al., 2011; Qin and Raymond, 2012; Zhang et al., 2017), only a handful—predominantly factors previously linked to stem cell reprogramming (Ralston and Rossant, 2010; Sterneckert et al., 2012) and/or retinal development (Lenkowski and Raymond, 2014)—have been investigated, let alone shown to be involved in eye repair (Goldman, 2014). Therefore, a central goal of this study was to leverage multiplexed CRISPR/Cas9-based mutagenesis (Shah et al., 2015; Varshney et al., 2015, 2016a) to enable a reverse genetic screen of hundreds of genes implicated in the regeneration of rod photoreceptor cells. Within the context of the current “reproducibility crisis” (Baker, 2016) large-scale discovery methods enable agnostic “hypothesis-generating” research that by nature eliminate investigator bias with respect to outcomes. Cloning-free sgRNA synthesis, multiplexing sgRNAs, and capillary electrophoresis evaluation of sgRNA activity make the CRISPR/Cas9 mutagenesis process relatively easy, fast and cost-efficient, facilitating large-scale mutagenesis efforts with a small team of postdoctoral fellows and technical support staff.

Multiplexing allows mutations to be induced at multiple target sites and assayed in tandem. Therefore, this approach is useful for labs that have limited aquaculture space. Multiplexing can also accelerate the observation of phenotypes that result from additive effects of several mutated genes (Shah et al., 2015). However, multiplexing increases toxicity and has been reported to reduce the mutagenic efficiency of sgRNAs (Jao et al., 2013). When pooling sgRNAs, caution must also be taken if the targets are on the same chromosome, as large deletions between target sites might occur. For instance, deletions of up to 1 Mb have been reported in zebrafish (Xiao et al., 2013). The loss of several genes or miRNA encoding sequences within a large deletion complicates analysis and should be avoided (Shah et al., 2015). On the other hand, when targeting genes for disruption, it can be advantageous to create deletions in key protein-coding domains to ensure that gene function is perturbed. Smaller, frame-shifting mutations can create false-negatives and cause the activation of a compensatory network to buffer against deleterious mutations (Rossi et al., 2015). Recent studies have suggested guidelines for researchers to ensure the generation of deleterious mutations (Ata et al., 2016; Anderson et al., 2017) and for precise genome editing in zebrafish (Hoshijima et al., 2016).

Our multiplexing strategy was designed to ensure a high rate of mutagenesis, with the goal of successfully investigating the role of each candidate gene in retinal regeneration. However, in the process of targeting two sites in each gene simultaneously we often observed small indels at both target sites rather than larger deletions of intervening regions. It is unclear if this is due to a limitation of the PCR assay in detecting larger deletions or a lack of the larger deletions being induced. Larger deletions were observed at only a ~10% rate in a related study (Varshney et al., 2015). Regardless, the possibility of having multiple mutations in the same gene in the F1 founder pool complicates the screening process, requiring additional effort to verify allele specificity prior to inbreeding (or verification of nonsense mediated decay of the mRNA). Interpretation of the role of specific mutant alleles on resulting phenotypes is otherwise not possible. On the other hand, recent reports detailing deleterious mutations and observable phenotypes occurring less frequently than expected as a result of mutagenesis techniques (Kettleborough et al., 2013; Anderson et al., 2017) emphasize the need to evaluate several mutant alleles per candidate gene. Thus, we still advocate designing multiple sgRNAs per targeted gene. However, unless the targets are contained within the same exon (i.e., intended to delete a key functional domain), we suggest multiplexing sgRNAs targeting different genes, residing on separate chromosomes, for pooled injections.

### Identification and phenotypic characterization of CRISPR/Cas9–induced mutants

For founder genotyping, we found both traditional agarose gel electrophoresis resolution of heteroduplex bands and capillary electrophoresis of fluorescent PCR products to be simple, cost-efficient, timely, and effective. The former is advantageous because most labs have the skills/equipment available. In addition, this method allows quick determination of injected mutation efficiency and visualization of larger deletions. The latter is advantageous because of the high-throughput nature of the analysis and the single base pair resolution that it offers when resolving smaller sized amplicons (~75–350 bp). Combining PCR samples that have been amplified with different universal fluorescently-labeled primers also allows the capillary electrophoresis analysis to be multiplexed (Ramlee et al., 2015; Varshney et al., 2015). A recent, alternative approach to founder screening involves high-throughput, next generation, amplicon sequencing of sperm samples from F0-injected fish (Brocal et al., 2016). This method allows for pre-screening for germline transmission and identification of individual F1 alleles while generating cryopreserved libraries of sperm samples at the same time.

A primary advantage of reverse genetics is the ability to genotype carriers, thereby enabling phenotyping in earlier filial generations than classic F3 forward genetics screens, and significantly reducing housing and husbandry burdens. Previous studies have shown that CRISPR/Cas9-based mutagenesis in zebrafish is amenable to phenotypic screening in the F0, F1, and F2 generations (Jao et al., 2013; Shah et al., 2015; Varshney et al., 2015). While screening CRISPR-injected F0 embryos allows for rapid phenotyping, genetic mosaicism complicates this analysis, necessitating highly robust and easily scored phenotypes. Similarly, although inbreeding F0-injected fish enables screening in the F1 generation, robust phenotypic assays are required due to non-Mendelian inheritance. Alternatively, genotyped F1 carriers can be inbred for F2 screens with predictable inheritance patterns. Regardless of which strategy is applied, each of these results in substantial savings in time, space and cost compared to traditional F3 screens.

To facilitate screening for retinal regeneration-deficient mutants we are inducing mutations in the *Tg(rho:YFP-NTR)gmc500* background. This transgenic line serves as a quick and quantifiable readout for the regeneration of rod photoreceptors following induction of selective rod cell ablation (Walker et al., 2012; White and Mumm, 2013; White et al., 2017). The degree of variability in the kinetics and number of photoreceptor cells regenerated post-ablation makes the analysis of mosaic F0 larvae or non-Mendelian F1 offspring impractical for this screen. Therefore, we are outcrossing founders with *rho:YFP-NTR* fish and analyzing regenerative capacity in the progeny of incrosses between F1 mutant carriers. In conjunction with our automated reporter quantification system (White et al., 2016), this strategy allows changes in cell loss and regeneration kinetics to easily be quantified, thereby facilitating the identification of developmental and regeneration-deficient mutant lines.

### CRISPR/Cas9-based retinal degenerative disease modeling

In mammalian model systems, numerous retinal disease models isolated as *de novo* mutations or generated with nuclease-targeting techniques exist. Still, CRISPR/Cas9-based mutagenesis provides a means of targeting the ever-increasing number of candidate genes associated with human retinal disease. For example, CRISPR/Cas9-edited mice homozygous for an RP-associated variant (p.Leu135Prp) in the *Receptor accessory protein 6* (*Reep6*) gene exhibit RP-like progressive photoreceptor degeneration and loss of visual function (Arno et al., 2016). In another example, postnatal lethality caused by CRISPR/Cas9-mediated disruption of the Leber's congenital amaurosis (LCA)-associated gene *potassium inwardly-rectifying channel, subfamily J, member 13* (*Kcnj13*) was circumvented by analyzing mosaic F0-generation mice. Loss of *Kcnj13* in retinal pigment epithelium (RPE) cells was associated with loss of overlying photoreceptors, suggesting *Kcnj13* functions in the RPE to maintain photoreceptor health (Zhong et al., 2015).

In contrast to mammalian model systems, the number of genetic retinal disease models in the zebrafish system is limited (Fadool and Dowling, 2008; Link and Collery, 2015; Stenkamp, 2015). In lieu of such resources, retinal degeneration studies largely rely on acute damage models (e.g., light ablation, puncture wounding, and drug-induced cell death). While useful for studying retinal regeneration, these methods fall short of providing adequate models for chronic diseases exhibiting retinal cell degeneration kinetics akin to human disease. CRISPR/Cas9-based mutagenesis methods can begin to fill this void by targeted disruption of, or introduction of specific mutations in, disease-linked genes. As an example, we present here a novel CRISPR/Cas9-generated zebrafish mutant allele of the gene *rho, rho*^*djh503*^, which exhibits an RP-like phenotype. Rhodopsin is a rod-specific G-protein coupled receptor composed of cytoplasmic, transmembrane and intradiscal domains. Mutations in *RHO* are responsible for approximately 30% of autosomal dominant RP, and 10% of autosomal recessive RP cases (Dias et al., 2017). More than 150 deleterious *RHO* mutations have been identified (The Human Gene Mutation Database, http://www.hgmd.cf.ac.uk/ac/index.php). The effects various mutations have on Rhodopsin function have been widely studied and categorized into six classes based on their biological consequences (Mendes et al., 2005). The *rho*^*djh503*^ mutation described here contains a 5 bp deletion close to the 3′ end of the *rho* gene predicted to fundamentally alter the protein's cytoplasmic tail. The location of the mutation in *rho*^*djh503*+/−^ and the dominant negative nature of its inheritance, most closely resembles class I mutations. These mutations are typically located at the 3′ end of the gene, do not affect protein folding, but impair transport from the inner to the outer segment of rod photoreceptors. An outer segment targeting signal is contained in a 44-amino acid region within the carboxy terminus (Tam et al., 2000). Disruption of this signal causes accumulation in the inner segment (Sung et al., 1994) which may activate aberrant calcium signaling (Sung and Tai, 2000) or interfere with the post-Golgi transport pathway (Green et al., 2000) to trigger rod cell death (Portera-Cailliau et al., 1994). Thus, the dominant negative effect of this mutation could be due to failed translocation of Rhodopsin to the outer segment, a possibility that warrants further investigation.

### CRISPR/Cas9-enabled mutagenesis screen for genes required for regeneration

Another primary goal of our study is to leverage multiplexed CRISPR/Cas9-based mutagenesis to enable a large-scale screen aimed at identifying genes required for retinal regeneration. Proof-of-principle for this approach was recently reported in a related guided mutagenesis screen for genes implicated in hair cell regeneration (Pei et al., 2018). In this study, a total of 254 mutations were screened to identify 7 genes required for hair cell replacement in lateral line neuromasts. All 7 genes were implicated in our transcriptomic analysis of retinal regeneration, though only one was included in our list of targeted genes, *hspd1*. Interestingly, with regard to the question of functional specificity, some but not all 7 genes were also required for either fin or liver regeneration. Studies comparing mutations across different cell-specific ablation paradigms will allow further exploration of functional specificity for genes linked to regeneration-deficient phenotypes.

## Summary

The genome modification strategies applied here allow mutational disease modeling and genetic screening to be carried out on a large scale using multiplexing of sgRNAs to target either single or multiple genes. We outline a readily applicable framework for CRISPR/Cas9-based screens for retinal regeneration mutants, establishing methods that can be used with other large-scale mutagenesis efforts. We anticipate that findings generated from this study will bring us closer to comprehensively interrogating the regulation of MG regenerative potential and providing treatments for patients with degenerative retinal diseases.

## Data availability statement

The raw data supporting the conclusions of this manuscript will be made available by the authors, without undue reservation, to any qualified researcher.

## Author contributions

AUE, TM, LZ, DW, SS, SB, MS, and JM designed experiments. AUE, TM, LZ, MS, and JM wrote the initial manuscript with subsequent contributions from all authors. AUE, TM, LZ, DW, SS, CN, NL, LX, and WP, performed experiments. AUE, TM, LZ, DW, SS, LX, WP, JQ, SB, and JM analyzed data.

### Conflict of interest statement

JM holds patents for the use of the nitroreductase (NTR) system in zebrafish regarding the identification of genes and chemical compounds that modulate regeneration. JM is the founder and MS is the president of Luminomics, a company developing the automated quantitative reporter *in vivo* screening platform for commercial distribution. JM and MS own stock in Luminomics. The remaining authors declare that the research was conducted in the absence of any commercial or financial relationships that could be construed as a potential conflict of interest.
