# Supplementary material for: Multiplexed CRISPR/Cas9 Targeting of Genes Implicated in Retinal Regeneration and Degeneration
Source: Front Cell Dev Biol. 2018 Aug 21;6:88. doi: 10.3389/fcell.2018.00088 (PMC6111214; doi:10.3389/fcell.2018.00088)
Supplement: Supplementary file 1 [file Image_1.pdf]

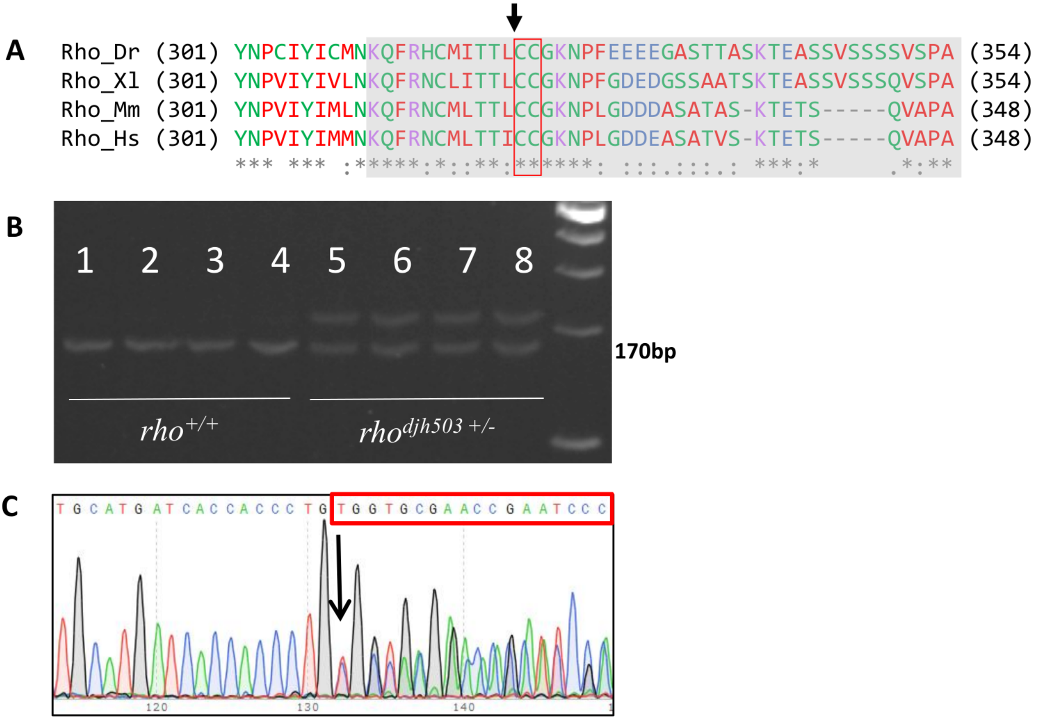

Supplemental Figure 1. Novel mutation in *rhodopsin* (*rho*), *rho*$^{djh503\ +/-}$. (**A**) Clustal Omega Alignment of the C-terminus of *Danio rerio* (*Dr*) Rhodopsin with the *Xenopus laevis* (*Xl*), *Mus musculus* (*Mm*) and *Homo sapien* (*Hs*) proteins. The terminal 44 amino acids necessary for outer segment localization are indicated by the grey box. Palmitoylated residues are boxed in red. The site of the frame shift mutation in *rho*^{djh503c} is marked by the arrow. (**B**) Agarose gel electrophoresis confirming the *rho* genotype for the larvae visualized in confocal studies. Heterozygous mutant amplicons also form a heteroduplex band that runs slower than the 170bp wildtype band. (**C**) Sanger sequencing of a PCR amplicon of *rho*$^{djh503\ +/-}$ gDNA. The site of the frame shift mutation is marked by the arrow. Altered sequence is boxed in red above the chromatogram.
