# Supplementary material for: Multiplexed CRISPR/Cas9 Targeting of Genes Implicated in Retinal Regeneration and Degeneration
Source: Front Cell Dev Biol. 2018 Aug 21;6:88. doi: 10.3389/fcell.2018.00088 (PMC6111214; doi:10.3389/fcell.2018.00088)
Supplement: Supplementary file 2 [file Image_2.pdf]

A

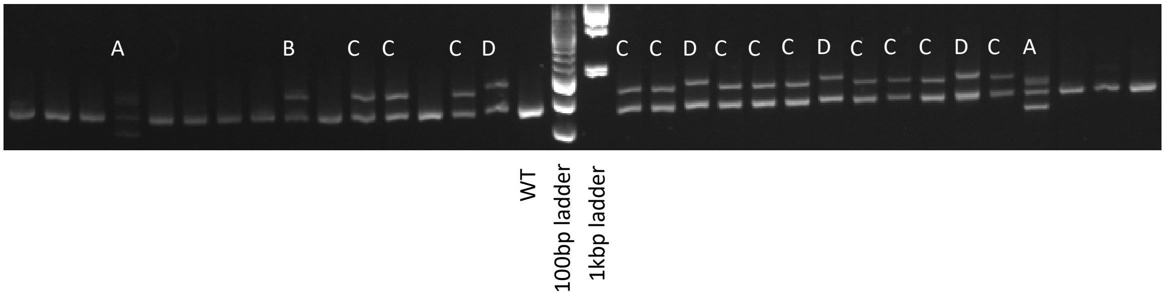

B

	Fish #	Size 1	Height 1	Size 2	Height 2	Size 3	Height 3
wt control 306 bp	1	x	x	x	x	306.13	7581
	2	x	x	x	x	306.15	9641
founder #1	1	282.29	5163	x	x	306.1	6394
	2	282.38	6787	x	x	306.04	6673
	3	x	x	x	x	306.11	9341
	4	x	x	298.17	2128	306.1	6834
	5	x	x	298.19	7105	306.12	4221
	6	282.38	7958	x	x	306.08	5071
	7	x	x	298.09	5286	306.05	4196
	8	282.36	6030	x	x	306.18	3660
	9	x	x	x	x	306.09	8375
	10	282.39	3947	x	x	306.12	4555

Supplemental Figure 2: **(A)** A representative example of *rho* mutant alleles transmitted to the F1 generation as analyzed by the formation of heteroduplex DNA on 3% agarose gel electrophoresis. Each separate allele has been designated by a letter above the corresponding lane. (A) = 42 bp deletion; (B) = 5 bp deletion; (C) = another 5 bp deletion; (D) = 4 bp insertion. **(B)** A representative example of the fluorescent PCR results genotyping an outcross of a mutant founder. Unique mutant alleles are shown in different colors. The wildtype amplicon size is 306bp. "Size" represents the length of the PCR fragment (in base pairs) when rounded to the nearest whole number. "Height" refers to the amplitude of the peak at the corresponding size.
