# Supplementary material for: Multiplexed CRISPR/Cas9 Targeting of Genes Implicated in Retinal Regeneration and Degeneration
Source: Front Cell Dev Biol. 2018 Aug 21;6:88. doi: 10.3389/fcell.2018.00088 (PMC6111214; doi:10.3389/fcell.2018.00088)
Supplement: Supplementary file 3 [file Image_3.pdf]

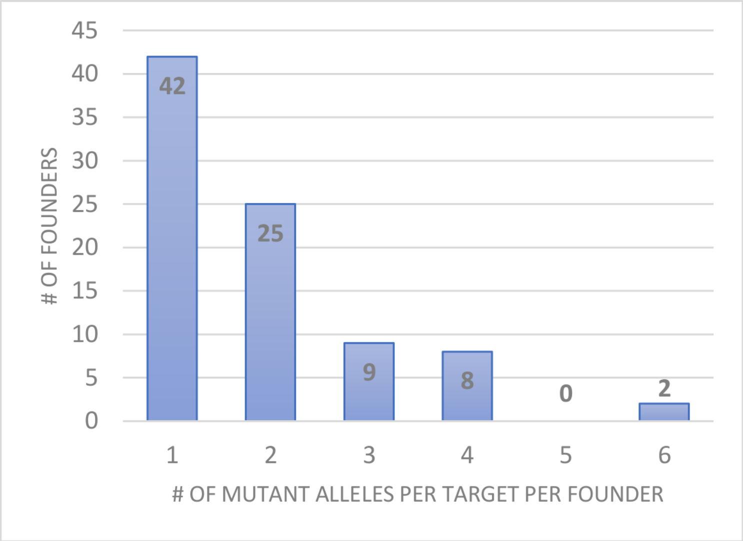

Supplemental Figure 3: Numbers of mutant alleles per assayed target seen in F1 progeny of founders. A minimum of 4 potential mutant larvae were assayed for each founder. Data only differentiates alleles based on size reported by capillary electrophoresis of fluorescent PCR amplicons and not actual sequence identity.
