# Supplementary material for: Multiplexed CRISPR/Cas9 Targeting of Genes Implicated in Retinal Regeneration and Degeneration
Source: Front Cell Dev Biol. 2018 Aug 21;6:88. doi: 10.3389/fcell.2018.00088 (PMC6111214; doi:10.3389/fcell.2018.00088)
Supplement: Supplementary file 4 [file Image_4.pdf]

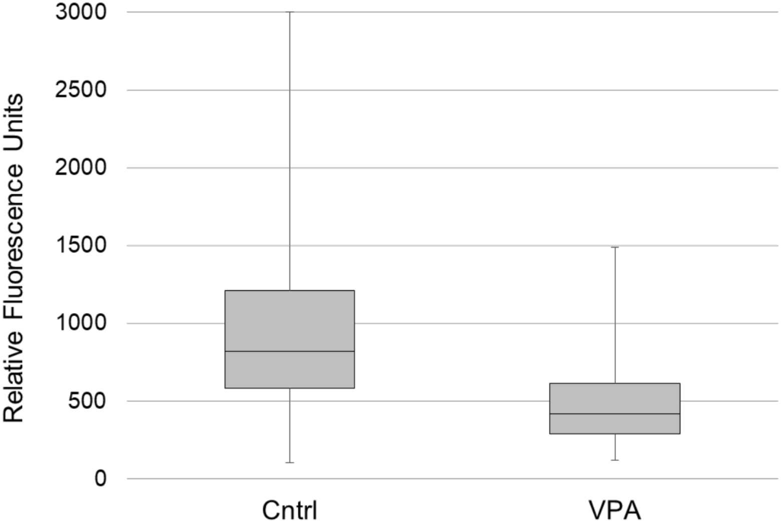

Supplemental Figure 4: Chemical inhibition of rod cell regeneration kinetics detected by ARQiv assay. Quantification of YFP signal intensity 3 days after induction of Mtz-meditated rod cell loss demonstrates valproic acid (VPA) treatment inhibition of rod cell regeneration kinetics. Control fish (Cntrl) subjected solely to rod cell loss have regenerated to ~50% of non-ablated YFP fluorescence levels by this time point. Conversely, VPA-treated larvae recover only ~25% of fluorescence.
