# Supplementary material for: Multiplexed CRISPR/Cas9 Targeting of Genes Implicated in Retinal Regeneration and Degeneration
Source: Front Cell Dev Biol. 2018 Aug 21;6:88. doi: 10.3389/fcell.2018.00088 (PMC6111214; doi:10.3389/fcell.2018.00088)
Supplement: Supplementary file 5 [file Image_5.pdf]

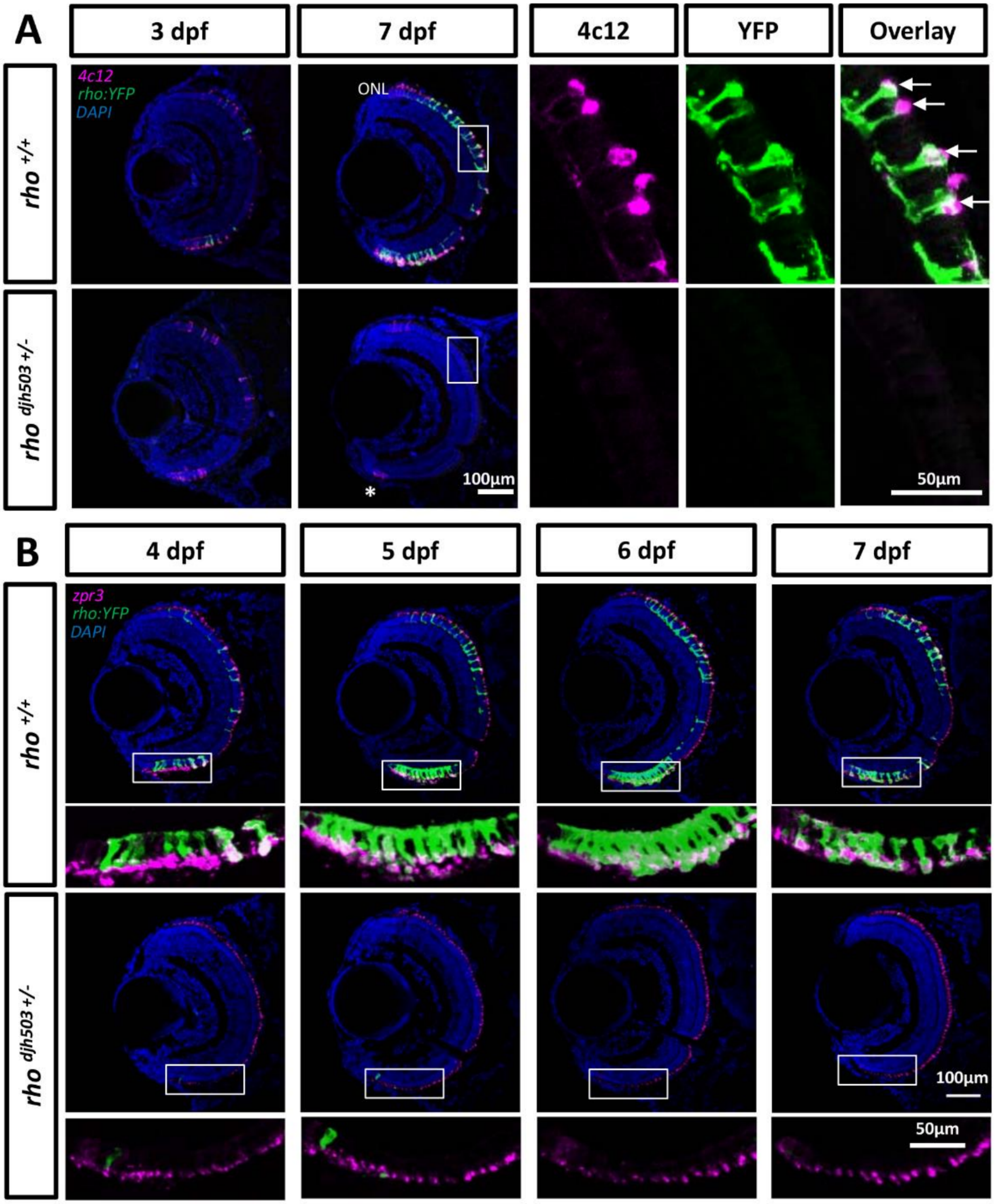

Supplemental Figure 5. Rod photoreceptor loss in *rho*$^{djh503\ +/-}$ mutants. **(A)** Anti-4c12 (magenta) labels rod cell membranes. The labeling pattern is similar in both wildtype and mutants at 3 dpf. At 7 dpf, anti-4c12 labeling is restricted to the proliferating marginal zone in *rho*$^{djh503\ +/-}$ mutants (asterisk), but remains throughout the ONL in wildtype retinas. The white boxed region in the 7 dpf images are enlarged in the panels to the right showing that anti-4c12 labels YFP-expressing rod cells (green, arrows). **(B)** The zpr3 (magenta) antibody labels both cone and rod photoreceptors. In *rho*$^{djh503\ +/-}$ mutants, few cells are positive for zpr3 antibody staining or YFP expression (green) at 4 or 7 dpf. The boxed ventral region of each image is enlarged below. DAPI (blue) was used to stain nuclei. Abbreviations: 4',6-diamidino-2-phenylindole (DAPI); days post-fertilization (dpf); outer nuclear layer (ONL); rhodopsin (rho); yellow fluorescent protein (YFP).
